# Reconstruction Considerations Following Complete Excision of Mucoid-Degenerated Anterior Cruciate Ligament: A Retrospective Study

**DOI:** 10.7759/cureus.53735

**Published:** 2024-02-06

**Authors:** Sashitemjen Aier, Anurag Das, Shalini Nayak, Vivek Pandey

**Affiliations:** 1 Orthopedics, Kasturba Medical College, Manipal, Manipal Academy of Higher Education, Udupi, IND; 2 Physiotherapy, Manipal College of Health Professionals, Manipal Academy of Higher Education, Udupi, IND

**Keywords:** instability, functional outcome, debridement, total excision, mucoid degeneration, anterior cruciate ligament

## Abstract

Introduction: Mucoid degeneration (MD) of the anterior cruciate ligament (ACL) is an unusual cause of knee pain and restricted movement, predominantly affecting the middle-aged population. Arthroscopic partial or total debridement of the mucoid ACL is the surgical treatment of choice. However, little is discussed in the literature regarding subsequent knee instability and functional outcomes following complete ACL excision.

Methods: A retrospective study was conducted on patients who underwent arthroscopic total ACL excision for mucoid ACL. Pre- and post-operatively, the Tegner-Lysholm score, the International Knee Documentation Committee (IKDC) Subjective Knee Form, and subjective functional instability were used to grade the clinical outcomes.

Results: Ten out of the 13 patients who underwent complete ACL excision were available for evaluation. All patients presented with knee pain on deep flexion or extension with a painfully limited range of motion. Post-operatively, all patients were relieved of their original pain and dysfunction. The mean post-operative IKDC and Tegner-Lysholm scores were 74.96 and 83.6, respectively. All patients had a Lachman test positive, while only two had a grade 1 pivot shift test positive. Two patients had occasional functional instability only after strenuous exercises. None of the patients underwent subsequent ACL reconstruction.

Conclusion: All patients reported improved functional outcomes. Only two out of 10 reported occasional instability during strenuous activity. Therefore, complete debridement of mucoid ACL in sedentary patients is safe and efficacious. However, active young patients may experience instability and require ACL reconstruction if it hinders their daily activities.

## Introduction

Mucoid degeneration (MD) of the anterior cruciate ligament (ACL) is characterized by ACL hypertrophy due to the deposition of glycosaminoglycans between the loose and thin collagen fibrils of the ligament [1]. MD of ACL is a condition of vacillating origin with numerous theories that hypothesize its geneses, such as the degenerative, ectopic synovial tissue, and microtrauma theories [2,3]. It is generally observed in the middle-aged population, varying between 35 and 60 years [4], who complain of insidious-onset vague knee pain and a terminally restricted range of motion. Notably, affected individuals often lack a history of significant trauma.

The majority of clinicians manage MD of ACL conservatively. Nevertheless, if it does not respond to conservative treatment, the current consensus in the literature for treating MD of ACL is arthroscopic debridement, partial or total [5-9]. While many authors advocate that debridement of mucinous tissue with partial ACL debulking is adequate without jeopardizing knee stability [7,8,10,11], a few authors have reported total excision of the ACL without any significant effect on functional knee stability [6,9,12].

Therefore, we aimed to investigate the post-operative functional outcomes and residual instability after the total excision of MD of ACL.

## Materials and methods

Patient selection

Following approval from Kasturba Medical College and Kasturba Hospital Institutional Ethics Committee (approval number: IEC/143/2023), a retrospective study was undertaken on individuals diagnosed with MD of ACL who had undergone arthroscopic "total ACL excision" from 2015 to 2021. The inclusion criteria encompassed patients aged 18 years and older who had undergone total excision of mucoid ACL with a minimum follow-up duration of one year. Patients with concurrent ligament injuries and fractures around the ipsilateral knee were excluded from the study. All the patients in the study underwent conservative treatment in the form of analgesics and physiotherapy for at least two to six months. Only those patients who had persistent symptoms after failed conservative treatment underwent surgical debridement.

Patient evaluation

The diagnosis of all patients with mucoid ACL was based on MRI findings, arthroscopic findings, and confirmation with biopsy. Functional outcomes were assessed using the Tegner-Lysholm score and the International Knee Documentation Committee (IKDC) Subjective Knee Score [13,14]. The preoperative and latest clinical follow-up scores were obtained from the individual case records stored in the electronic database of the Medical Records Department. Furthermore, all patients were asked if they experienced instability in the operated knee during daily activities or sports at the one-year or final follow-up. This data was interpreted as indicative of subjective functional instability.

Operative treatment

A single senior surgeon performed the surgical procedure on all patients. Before the surgery, all patients were informed regarding partial or total debridement of the ACL, depending on the amount of mucoid change in the ACL. They were informed that a total debridement might result in knee instability. However, being sedentary in activity, no patient consented to primary ACL reconstruction and opted for second-stage ACL if instability arose, affecting their activities of daily living.

Diagnostic arthroscopy was performed from the standard anterolateral and anteromedial portals, and the condition of the menisci, ligaments, and cartilage was noted. The concomitant cartilage lesions were graded per the International Cartilage Research Society (ICRS) classification and treated according to standard protocol [15]. ICRS grade I-III underwent debridement, whereas microfracture was performed for ICRS grade IV lesions. Patella cartilage lesions were only debrided, and no microfracture was performed. All the meniscal lesions were degenerative and complex and, therefore, were debrided. No meniscal repairs were performed. In all cases, the posterior cruciate ligament was found to be intact. The ACL was probed for the presence of mucoid changes within the substance. In all cases, the intercondylar notch was found to be stuffed with a thickened mucoid ACL, with some instances having cysts at the base of the ACL on the tibial attachment site.

The arthroscopic debridement for the MD of ACL was performed using a 4.0 mm motorized shaver and/or a radiofrequency ablation device. The partial or total debridement of mucoid ACL was based on the number of fibers involved in the mucoid process. If at least 30-40% of ACL fibers appeared uninvolved after partial debridement, they were left in place. However, if more than 70-80% of ACL fibers were involved in the mucoid process, the entire ACL would be excised. Notchplasty was not done in any case, as the complete removal of the ACL ensured adequate space in the notch. No ACL reconstruction was performed in any case. During debridement, biopsy specimens were taken from mucoid ACL using basket forceps, which were sent for histopathological examination.

Post-operative rehabilitation

All patients were mobilized from the first post-operative day, and full-weight bearing was allowed without any brace. All patients underwent standard knee rehabilitation, strengthening the hamstring and quadriceps and achieving complete flexion. Post-operatively, all patients were followed up at one, three, six, and twelve months.

Statistical analysis

SPSS Statistics version 23 (IBM Corp. Released 2015. IBM SPSS Statistics for Windows, Version 23.0. Armonk, NY: IBM Corp.) was used for data analysis. Descriptive statistics were used to assess means, standard deviations, and percentages. The Wilcoxon-Mann-Whitney-Mann-Whitneypplied to compare pre- and post-operative clinical scores.

## Results

From 2015 to 2021, 28 patients were operated on for mucoid ACL. Among 28 patients, a total of 13 underwent complete excision of the mucoid-degenerated ACL and a remaining partial excision. Three patients who underwent a complete ACL excision were lost to follow-up. Thus, a total of 10 patients were available for evaluation. There were five (50%) males and five (50%) females. The mean (±SD) age at the time of surgery was 42.9 (±9.12) years. The mean (±SD) of the follow-up duration was 51.2 (±23.17) months. The baseline details of all the patients are mentioned in Table 1. All patients followed a sedentary lifestyle; none were recreational or professional athletes.

**Table 1:** Baseline demographic, clinical features, MRI, arthroscopy finding, and pre- and post-operative Lysholm and IKDC score of all patients M: male, F: female, R: right, L: left, ACL: anterior cruciate ligament, MM: medial meniscus, LM: lateral meniscus, MFC: medial femoral condyle, LFC: lateral femoral condyle, MD: mucoid degeneration. The starred serial number numbers (3 and 5) are the patients who experienced instability after strenuous activity

S. no.	Age/sex	Side	Duration of symptoms (months)	Symptoms	MRI findings (other than mucoid ACL)	Arthroscopy findings with respect to meniscus and cartilage	Pre-op IKDC/Lysholm score	Post-op IKDC/Lysholm score	Follow-up (months)
1	42/F	L	24	Pain, painful deep flexion	Complex MM posterior third tear	Complex MM posterior third tear, grade 2 cartilage defect over MFC	34.5/61	71.3/82	91
2	31/M	R	6	Pain, painful deep flexion	MD of ACL with ganglion cyst and intraosseous cysts under the base of ACL	Grade 1 MFC cartilage defect	27.6/53	75.9/95	75
3	37/M*	R	12	Pain, locking, and clicks	Horizontal tear of the MM	Complex tear of middle third MM, fraying of the anterior and posterior third of LM	31/67	79.3/84	81
4	34/M	L	72	Pain, painful terminal extension	A few small cysts were seen adjacent to the ACL	Normal	47.1/81	93.1/95	31
5	33/F*	L	4	Pain, painful deep flexion	MD of ACL with adjacent ganglion cyst. Tear in the anterior horn of lateral meniscus with parameniscal cyst	MM posterior third degenerative tear, cyst over the anterior horn of MM	43.7/65	69/59	51
6	46/M	R	24	Pain, painful deep flexion	MD of ACL	Grade 4 cartilage defect over MFC and grade 2 over LFC	39.1/67	82.8/100	46
7	53/F	L	2	Pain, painful terminal extension	Horizontal tear of medial meniscus. Cartilage lesions over the medial and lateral patellar facet	Grade 4 cartilage defect over MFC, Chondromalacia patella	26.4/59	83.9/100	42
8	57/M	R	6	Pain, painful deep flexion	Longitudinal tear in the posterior horn of the medial meniscus. Chondropathic changes in retropatellar cartilage	Grade 4 cartilage defect over MFC, complex tear of posterior third MM, chondromalacia patella	47.1/67	59.8/70	41
9	52/F	L	6	Pain, clicking sensation	Mucoid ACL, MM posterior third complex tear	Posterior third MM complex tear, grade 3 MFC cartilage defect, chondromalacia patella, cyst over ant horn of lateral meniscus	34.5/61	73.6/85	27
10	44/F	R	24	Pain, locking, and clicks	Mucoid ACL, cyst at the base of ACL	Cyst at ACL base, Grade 3 cartilage defect over MFC and trochlea	27.6/53	60.9/66	27

Preoperatively, all 10 patients presented with pain during activities and movement. Five patients had flexion deficits varying between 10° and 30° compared to normal knees, whereas two patients had a 5° extension deficit. The mean (±SD) preoperative IKDC and Tegner-Lysholm scores were 35.28 (±8.33) and 63.60 (±8.26), respectively.

Post-operatively, all patients regained full range of movement. The mean (±SD) post-operative IKDC score and Tegner-Lysholm score at the latest follow-up were 74.96 (±10.37) and 83.6 (±14.54), respectively. The preoperative to post-operative change in IKDC and Tegner Lysholm scores was statistically significant (Table 2).

**Table 2:** Comparing the means (±SD) of pre- and post-operative IKDC and Tegner-Lysholm scores. Significance was kept at p<0.05

	Preoperative	Post-operative	p-value
IKDC	35.28 (±8.33)	74.96 (±10.37)	0.006
Tegner-Lysholm score	63.60 (±8.26)	83.60 (±14.54)	0.014

At the final follow-up, all patients had a positive Lachman test ranging from grade 1 to 2. Only two patients had a grade 1 positive pivot test (S. no. 3 and 5 in Table 1) and also perceived functional instability while walking for a prolonged duration or doing strenuous activity. However, they felt that the occasional instability wasn’t disrupting their daily routine. Therefore, they did not opt for ACL reconstruction.

## Discussion

Our study demonstrated that total excision of the mucoid ACL in a sedentary, non-athletic population results in improved functional outcomes. Furthermore, not all patients experience subjective instability despite having an ACL-deficient knee.

After the unmasking of the condition of MD of ACL by Kumar et al., the condition was considered a rare pathological entity [1]. Nevertheless, Bergin et al. and Salvati et al. revealed that it is far more common than reported [16,17]. Bergin et al. retrospectively studied 4,221 consecutive knee MRIs and reported MD in 74 (1.8%) knees, 24% had pure MD, and 76% had mucoid cysts [16]. Similarly, Salvati et al. studied 1215 knee MRIs and found 64 (5.3%) knees had MD [17].

The pathogenesis of MD remains idiopathic. One theory postulates that repetitive activities could lead to the traumatic disruption of ACL fibers with insufficient repair, subsequently resulting in the organization of mucoid material [18]. Alternative theories depict MD and ganglion cyst formation as entities along a continuum of connective tissue mucinous degeneration, herniation of synovial tissue, ectopia of synovial tissue, and proliferation of pluripotential mesenchymal stem cells as a cause of mucinous degeneration [16,19]. While these theories postulate the origin or cause of the MD of the ACL, we believe that the MD is part of the whole degeneration of the knee. This may be supported, as 90% of our patients had associated cartilage lesions and complex degenerative-type meniscal tears. Himpe et al. also reported similar findings in their study [12]. They had retrospectively studied 11 knees that underwent total excision of mucoid-degenerated ACL and noted that 10 knees had medial compartment arthritis and eight had a medial meniscus tear [12]. Many other bony factors are also implicated in the pathogenesis of the mucoid ACL, such as increased posterior slope, decreased notch width index, increased tibial tuberosity-trochlear groove distance, and trochlear dysplasia [20]. However, we have not assessed any of those bony factors as several MRIs were not performed in our hospital, limiting the analysis. Moreover, the analysis of bony factors was not the aim of our study.

MD of the ACL generally presents posterior knee pain [2,21]. It is theorized that posterior knee pain arises due to increased pressure in the intercondylar notch and the posterior capsule [2,22]. In many cases of symptomatic mucoid ACL, the ACL is noted to be homogenously hypertrophied, filling up the entire intercondylar notch, which may explain the reduction of the range of movement and extension block. Most studies indicate that during clinical examination, a restricted range of motion is typically observed in flexion, approximately 100° [1,8,21]. In other studies, some patients present with limited and painful extension [2]. In our research, we encountered both entities; several patients presented with a flexion deficit between 10° and 30°, while 20% presented with an extension deficit.

An MRI of the knee is diagnostic in cases where a mucoid ACL is suspected. The classic findings of mucoid ACL include bulky and ill-defined ACL and increased intra-ligamentous signals (intermediate signal intensity on T1-weighted images, high signal intensity on T2-weighted images) with the celery stalk appearance [23]. However, overall, ACL fibers remain intact [8]. It is crucial to highlight that MD of ACL is frequently misidentified on MRI scans as a partial ACL rupture [8,23]. Further, using Bergin’s criteria, one must differentiate a mucoid ACL from a mucoid cyst [16,23]. Mucoid cysts have a mass effect on ACL fibers, lobulated margins, fluid signals within the substance of the ACL, and intact ACL fibers. The arthroscopic diagnostic criteria for mucoid ACL include (1) the presence of continuous ACL fibers, (2) an augmented ACL volume, (3) yellowish-colored material expressed upon palpation or interspersed between ACL fibers, and (4) the absence of ACL synovial lining. Mucoid cysts appear as separate lobulated lesions within the ACL substance or the tibial or femoral attachment points [23]. Histologically, a mucoid substance in ACL connective tissue contains glycoproteins and mucoproteins [22]. The differentiation between the intra-ACL mucoid cyst and mucoid ACL is important, as the former condition requires selective cyst debridement, whereas the latter requires partial or total ACL debridement.

A wide array of treatments have been described in the current literature, ranging from partial to total excision of the ACL with or without notchplasty [2,5,6,8,9,12,22,24,25]. A conclusion drawn from a systematic review conducted by Vivekanantha et al. suggests that most authors believe in a non-aggressive method of partial ACL debridement, as there has not been any report of recurrence, and the remaining fibers may prevent instability [11]. Notchplasty, along with partial debridement, was denounced by several authors, who believed that a thorough debridement of the bulky ACL would remove the impingement [8,19]. Furthermore, notchplasty is unquestionably unnecessary if the ACL is completely debrided.

A partial ACL excision is justified if some fibers of the ACL are spared from MD or if only one of the two bundles is involved. However, total excision of the ACL becomes imminent if the mucoid process involves almost the entire ACL.

While the literature is replete with outcomes after partial ACL debridement, only a few authors have reported the clinical outcome after total ACL excision [6,9,12,25]. Nevertheless, the overall clinical outcome, sans instability, in the systematic review by Vivekanantha et al. is comparable in the groups that underwent partial vs. total ACL excision [11].

In their report, Lintz et al. documented a complete excision of the ACL in 17 out of 29 patients diagnosed with MD [6]. They defined "partial excision" as when less than 50% of fibers were excised, whereas "total excision" is the debridement of more than 50% of ACL fibers [6]. In our study, we considered the total removal of all ACL fibers as total excision. Even if a few strands were left, we considered it an incomplete excision. Although all authors report a high incidence of objective instability in the form of a positive Lachman test in the patients who underwent total ACL excision, the rate of subjective instability in those patients with positive Lachman was relatively low, varying from 0-11.7% [6,9,12,25]. Furthermore, very few patients with total ACL excision need to undergo ACL reconstruction, implying that not everyone will develop symptomatic instability despite having an ACL-deficient knee.

Lintz et al. reported ACL reconstruction in two out of 17 patients (11.7%), whereas Ventura et al. reported ACL reconstruction in only one patient in 18 (5.5%) who underwent total ACL reconstruction [6,9]. In contrast, Himpe et al. and Oehler et al. did not report any subjective instability or ACL reconstructions despite objectively unstable knees [12,25]. Table 3 summarizes these studies.

**Table 3:** All studies in the literature reported total excision of MD of ACL Lintz et al. had a total of 29 knees, of which 17 underwent total ACL excision. Ventura et al. had a total of 25 patients; seven underwent partial and 18 underwent total excisions of ACL. Himpe et al. had a total of 13 cases with total ACL excision out of which only 11 were followed up clinically. Oehler et al. had 20 cases, 10 each in partial and total excision groups. Lintz et al. did not categorize the results between partial and total excision. They had 29 cases (17 total excisions, 12 partial excisions) ACL-R: anterior cruciate ligament reconstruction done due to disabling instability, N/A: data not available

Study	Number of cases of total excision	Pre-op Lachman test	Pre-op pivot shift test	Post-op positive Lachman test	Post-op positive pivot shift test	Subjective instability	ACL-R performed
Lintz et al. (2010) [6]	17	1 (delayed hard stop)	0	16	8	2	2
Ventura et al. (2018) [9]	18	0	0	18	0	1	1
Himpe et al. (2020) [12]	13	3	N/A	3	N/A	0	0
Oehler et al. (2022) [25]	10	0	N/A	10	N/A	0	0

All our patients were relieved symptomatically and had regained full range of motion. Furthermore, all had resumed their prior levels of daily activities, but all were sedentary without engaging in sporting habits, thus having a less demanding ACL function. Furthermore, 80% (eight out of 10) of our patients did not experience any subjective instability. Although two patients experienced instability, they refused to undergo ACL reconstruction as the instability was occasional during strenuous work.

The pertinent question is why, despite complete ACL debridement, most of these patients do not develop functional instability. Though there is no concrete evidence, it can be postulated that several mechanisms might help prevent instability: an intact anterolateral ligament (ALL) and the recruitment of hamstrings during rehabilitation. ALL is recognized as a secondary stabilizer for anterolateral translation, primarily coming into play following the loss of ACL rather than serving as a co-stabilizer [26]. A cadaveric study conducted by Tavlo et al. demonstrated that detaching the ALL significantly impacted anteroposterior and internal rotation stability in knees devoid of the ACL [27]. They also reported that an intact ALL confers anteroposterior and rotational stability in an ACL-deficient knee [27]. However, the presence of ALL in the knee itself is under investigation. In cadaveric studies with similar dissection protocols, the prevalence of ALL is found to vary from 45.5% to 100% [28]. Furthermore, in an MRI and arthroscopy evaluation of 26 patients with acute ACL tears, Monaco et al. concluded that ALL/capsule injury was present in 88% of patients [29]. In a systematic review comprising 24 studies, Andrade et al. concluded that ALL is present in 51-100% of all knees and is injured in 11-79% of all ACL-injured knees [30].

However, considering the presence of ALL in most cases, surgical debridement of the mucoid ACL, partial or complete, leaves an intact ALL and other secondary stabilizers, which may give adequate stability to the knee for daily activities. However, the absence of ACL might affect sporting performance [31]. According to a meta-analysis by Filbay et al., ACL-deficient individuals exhibited health-related quality of life scores on average comparable to those of the general population. However, these scores were lower than those reported for individuals actively engaged in sports [32].

Furthermore, aggressive physiotherapy in the post-operative period may lead to the conditioning of the hamstrings and quadriceps, thereby overcoming the deficiency of the ACL. It is important to note that our two patients experienced “instability” only while performing strenuous activity. We can, therefore, hypothesize that the instability may occur when the hamstrings or quadriceps are fatigued. Given the unclear guidelines and unpredictability of instability after total ACL resection in mucoid ACL, more biomechanical and multicentric prospective clinical trials must be conducted to have more exacting guidelines regarding the management, especially who must be offered primary ACL reconstruction after total or near total excision of mucoid ACL.

Limitations of the study

One of the significant limitations of our study is its retrospective nature and inherent bias. The second shortcoming is the limited sample size. However, fewer cases are attributed to the fact that most cases can be managed with partial resection of mucoid ACL, and only a few require total excision. Thirdly, our series has no patients involved in athletics or sports. Therefore, we cannot compare ACL deficiency in patients with high demand vs. those who remain sedentary in activity.

## Conclusions

Complete excision of mucoid ACL is an efficacious procedure and does not result in subjective instability in most patients, especially those with a sedentary lifestyle. Nevertheless, certain younger patients enthusiastic about engaging in athletic pursuits may necessitate primary ACL reconstruction or, in the future, if they experience instability.
